# Supplementary material for: Durvalumab plus tremelimumab for the treatment of patients with progressive, refractory advanced thyroid carcinoma: the phase II GETNE-DUTHY trial
Source: Nat Commun. 2026 Apr 4;17:4891. doi: 10.1038/s41467-026-71155-y (PMC13230698; doi:10.1038/s41467-026-71155-y)
Supplement: Supplementary file 1 — Supplementary Information [file 41467_2026_71155_MOESM1_ESM.pdf]

A phase II study of durvalumab plus tremelimumab for the treatment of patients with progressive, refractory advanced thyroid carcinoma - The GETNE-DUTHY trial.

SUPPLEMENTARY FIGURES

Supplementary Figure 1. Kaplan–Meier curves stratified according to different baseline characteristics for each cohort. DTC cohort: a) PFS of patients with ECOG 0 (red) and ECOG 1 (blue-green) (p=0.056). **b)** OS of patients with ECOG PS 0 (red) and ECOG PS 1 (blue-green) (p=0.025). **MTC cohort: c)** PFS of female (red) and male (blue-green) patients (p=0.089) and **d)** OS of female (red) and male (blue-green) patients (p=0.108). **ATC cohort: e)** PFS of female (red) and male (blue-green) patients (p=0.336) and **f)** OS of female (red) and male (blue-green) patients (p=0.056). Two-sided log-rank test was used. Source data is provided as a Source Data file.

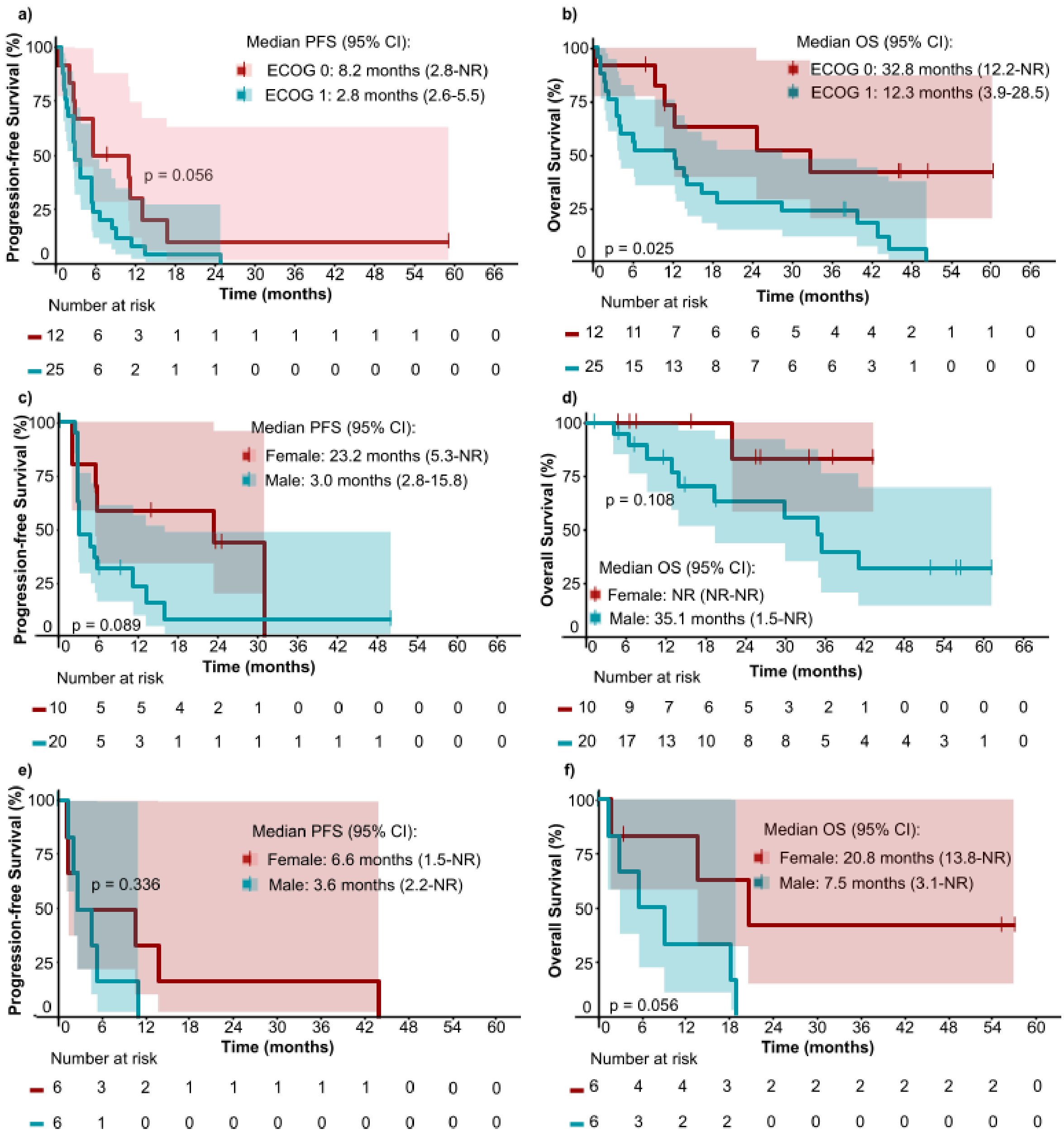
a)
Progression-free Survival (%)
Median PFS (95% CI):
ECOG 0: 8.2 months (2.8-NR)
ECOG 1: 2.8 months (2.6-5.5)
p = 0.056
Time (months)
Number at risk
12 6 3 1 1 1 1 1 1 1 0 0
25 6 2 1 1 0 0 0 0 0 0 0
b)
Overall Survival (%)
Median OS (95% CI):
ECOG 0: 32.8 months (12.2-NR)
ECOG 1: 12.3 months (3.9-28.5)
p = 0.025
Time (months)
Number at risk
12 11 7 6 6 5 4 4 2 1 1 0
25 15 13 8 7 6 6 3 1 0 0 0
c)
Progression-free Survival (%)
Median PFS (95% CI):
Female: 23.2 months (5.3-NR)
Male: 3.0 months (2.8-15.8)
p = 0.089
Time (months)
Number at risk
10 5 5 4 2 1 0 0 0 0 0 0
20 5 3 1 1 1 1 1 1 0 0 0
d)
Overall Survival (%)
p = 0.108
Median OS (95% CI):
Female: NR (NR-NR)
Male: 35.1 months (1.5-NR)
Time (months)
Number at risk
10 9 7 6 5 3 2 1 0 0 0 0
20 17 13 10 8 8 5 4 4 3 1 0
e)
Progression-free Survival (%)
p = 0.336
Median PFS (95% CI):
Female: 6.6 months (1.5-NR)
Male: 3.6 months (2.2-NR)
Time (months)
Number at risk
6 3 2 1 1 1 1 1 0 0 0
6 1 0 0 0 0 0 0 0 0 0
f)
Overall Survival (%)
Median OS (95% CI):
Female: 20.8 months (13.8-NR)
Male: 7.5 months (3.1-NR)
p = 0.056
Time (months)
Number at risk
6 4 4 3 2 2 2 2 2 2 0
6 3 2 2 0 0 0 0 0 0 0

Supplementary Figure 2. Swimmer plot of all included patients divided by cohort. Time-line representation of treatments, principal events, and follow-up of patients with DTC (red), MTC (blue-green) and ATC (yellow). The solid inner line shows the treatment duration and the shaded clear area shows the follow-up duration. Source data is provided as a Source Data file. * Patients with ATC who received radiotherapy treatment prior to inclusion.

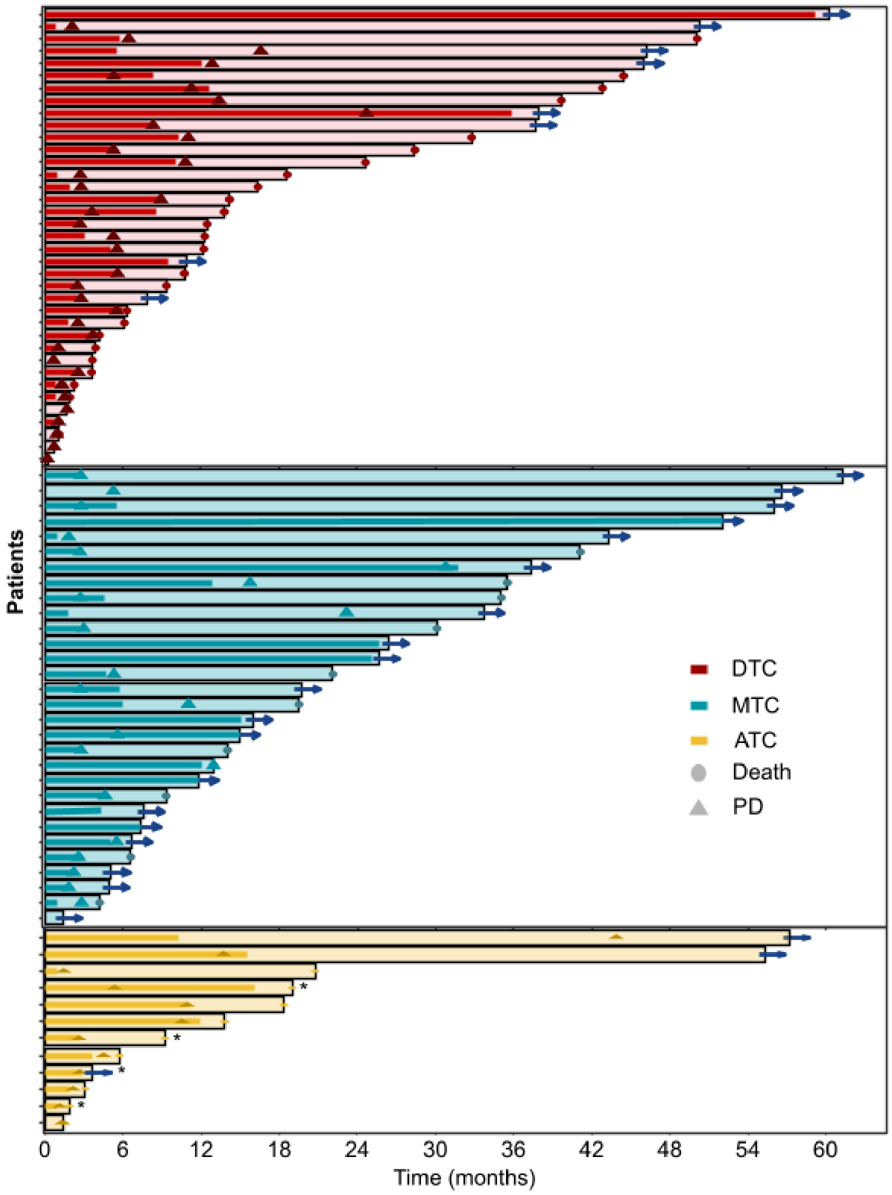

Supplementary Figure 3. Kaplan-Meier curves for all included patients stratified by the presence or not of liver metastases (LM). a) Progression-free survival of patients with (blue-green) and without (red) liver metastasis (p=0.872). **b)** Overall survival of patients with (blue-green) and without (red) liver metastasis (p=0.977). Two-sided log-rank test was used. Source data is provided as a Source Data file.

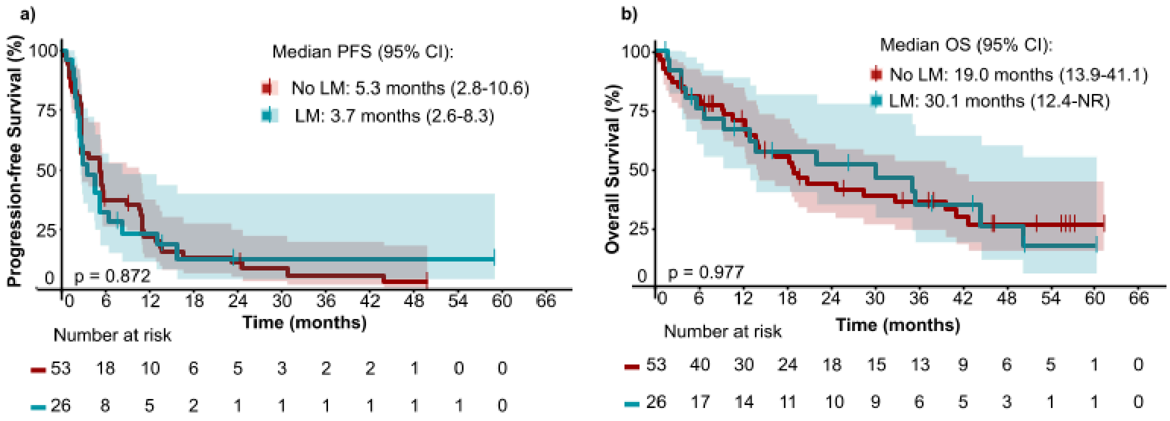

Supplementary Figure 4. Trial scheme. Schematic of study treatments, from the time informed consent was signed to the end of the study.

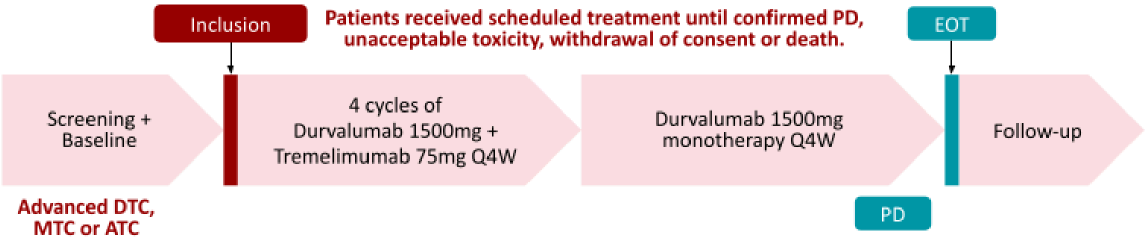

SUPPLEMENTARY TABLES

Supplementary table 1. Estimated progression-free survival for the three cohorts at 6, 12 and 18 months. Source data is provided as a Source Data file.

PFS	Events (%, total N)	Patients at risk	% estimated cumulative survival rate	CI 95%
**DTC**				
**6 months**	25 (67.6, 37)	12	32.4	(20.4 , 51.6)
**12 months**	31 (83.8, 37)	5	14.9	(6.7 , 32.9)
**18 months**	34 (91.9, 37)	2	6.0	(1.6 , 22.6)
**MTC**				
**6 months**	17 (56.7, 30)	10	40.9	(26.3 , 63.6)
**12 months**	18 (60.0, 30)	8	36.3	(22.1 , 59.8)
**18 months**	20 (66.7, 30)	5	26.5	(13.6 , 51.6)
**ATC**				
**6 months**	8 (66.7, 12)	4	33.3	(15.0 , 74.2)
**12 months**	10 (83.3, 12)	2	16.6	(4.7 , 59.1)
**18 months**	11 (91.7, 12)	1	8.3	(1.3 , 54.4)

Supplementary table 2. Estimated overall survival for the three cohorts at 6, 12 and 18 months. Source data is provided as a Source Data file.

OS	Events (%, total N)	Patients at risk	% estimated cumulative survival rate	CI 95%
**DTC**				
**6 months**	11 (29.7, 37)	26	70.3	(57.0 , 86.7)
**12 months**	15 (40.5, 37)	20	59.2	(45.3 , 77.5)
**18 months**	21 (56.8, 37)	14	41.5	(28.0 , 61.4)
**MTC**				
**6 months**	1 (3.3, 30)	26	96.6	(90.1 , 100)
**12 months**	3 (10.0, 30)	20	88.6	(77.2 , 100)
**18 months**	5 (16.7, 30)	16	79.8	(65.3 , 97.5)
**ATC**				
**6 months**	4 (33.3, 12)	7	65.6	(43.2 , 99.8)
**12 months**	5 (41.7, 12)	6	56.3	(33.6 , 94.3)
**18 months**	6 (50.0, 12)	5	46.9	(25.0 , 87.9)

Supplementary table 3. Comparison between the best overall response assessed by RECIST 1.1 and irRECIST 1.1. The agreement among RECIST and irRECIST was assessed using two-sided Cohen's kappa test, showing a statistically significant concordance ($p<0.05$). Source data is provided as a Source Data file.

Best OR (RECIST)	PD (n=27)	PR (n=10)	SD (n=36)	UK (n=6)	Total (n=79)	p-value
**Best OR (irRECIST)**						p<0.001; [0.924 (0.852 ; 0.995)][1]
CR	0 (0.0%)	1 (10.0%)	0 (0.0%)	0 (0.0%)	1 (1.3%)	
PR	0 (0.0%)	9 (90.0%)	0 (0.0%)	0 (0.0%)	9 (11.4%)	
SD	1 (3.7%)	0 (0.0%)	36 (100.0%)	0 (0.0%)	37 (46.8%)	
PD	24 (88.9%)	0 (0.0%)	0 (0.0%)	0 (0.0%)	24 (30.4%)	
NE	2 (7.4%)	0 (0.0%)	0 (0.0%)	0 (0.0%)	2 (2.5%)	
UK	0 (0.0%)	0 (0.0%)	0 (0.0%)	6 (100.0%)	6 (7.6%)	

1. Cohen's kappa p-value; [Estimate (CI 95%)]

Supplementary table 4. Subsequent treatments received by patients with a survival time of 4 years or more who progressed to the study treatment. The listed are independent lines of treatment. Source data is provided as a Source Data file.

	DTC n=3	MTC n=4	ATC n=2
Blinded clinical trial	1 (33%)	1 (25%)	-
Cabozantinib	1 (33%)	1 (25%)	-
Lenvatinib	2 (67%)	-	-
Selpercatinib	-	2 (50%)	-
Carboplatin	-	-	1 (50%)
Taxol	1 (33%)	-	1 (50%)
Gemcitabine	-	-	1 (50%)
Pegylated liposomal doxorubicin	-	-	1 (50%)
Radiotherapy	1 (33%)	-	-
No subsequent treatment	-	-	1 (50%)

Supplementary table 5. Grade of most frequent treatment-related adverse events (TRAEs). Source data is provided as a Source Data file.

TRAE	G1-2	G3-4	Overall
**Dermatologic events**; n (%)	35 (44.3)	0 (0)	35 (44.3)
**Fatigue**; n (%)	14 (17.7)	2 (2.5)	16 (20.3)
**Diarrhea**; n (%)	11 (13.9)	1 (1.3)	12 (15.2)
**Hepatic enzyme elevated**; n (%)	8 (10.1)	1 (1.3)	9 (11.4)
**Lipase increased**; n (%)	4 (5.1)	1 (1.3)	5 (6.3)
**Serum amylase increased**; n (%)	3 (3.8)	1 (1.3)	4 (5.1)
**Dyspnea**; n (%)	1 (1.3)	1 (1.3)	2 (2.5)
**Fever**; n (%)	1 (1.3)	0 (0)	1 (1.3)

Supplementary table 6. Grade of most frequent immune mediated adverse events (imAE) with a 5% threshold overall. Source data is provided as a Source Data file.

**imAE**	**G1-2**	**G3-4**
**Rash,** n (%)	13 (16.5)	0 (0)
**Diarrhea,** n (%)	10 (12.7)	1 (1.3)
**Dermatitis,** n (%)	7 (8.9)	0 (0)
**Hepatic enzyme elevated,** n (%)	6 (7.6)	0 (0)
**Lipase increased,** n (%)	4 (5.1)	1 (1.3)
**Serum amylase increased,** n (%)	3 (3.8)	1 (1.3)

Supplementary table 7. Estimation of primary endpoints using binomial calculations.

		total N	Events	6-month rate, %	CI 95%, %
**DTC**	**PFS**	37	25	32.4	18.0-49.8
	**OS**	37	11	70.3	53.0-84.1
**MTC**	**PFS**	27	17	37.0	19.4-57.6
	**OS**	27	1	96.3	81.0-99.9
**ATC**	**PFS**	12	8	33.3	9.9-65.1
	**OS**	11	4	63.6	30.8-89.1
