# Supplementary material for: Durvalumab plus tremelimumab for the treatment of patients with progressive, refractory advanced thyroid carcinoma: the phase II GETNE-DUTHY trial
Source: Nat Commun. 2026 Apr 4;17:4891. doi: 10.1038/s41467-026-71155-y (PMC13230698; doi:10.1038/s41467-026-71155-y)
Supplement: Supplementary file 2 — Description of Additional Supplementary Files [file 41467_2026_71155_MOESM2_ESM.pdf]

Description of Additional Supplementary Files

Supplementary Data 1: List of known molecular profiles and the techniques used for the determination. The molecular profile was not routinely assessed. The data are available only if they were assessed following local standard procedures.
